# Effect of zinc on cadmium induced oxidative stress: an in vivo study

**DOI:** 10.1186/1939-4551-8-S1-A258

**Published:** 2015-04-08

**Authors:** Ibrahim Alhazza

**Affiliations:** 1 King Saud University, Saudi Arabia

## Background

The effects of Cadmium (Cd) exposure and the treatment with Zinc (Zn) on hepatic oxidative stress was studied.

## Methods

The exposure of rats to Cd was at a dose of 2.2 mg/kg CdCl_2_, injected subcutaneously four times weekly for 2 months. Rats were supplemented with Zn (2.2 mg/kg ZnCl_2_, injected subcutaneously four times weekly for 2 months) one hour prior to Cd exposure.

## Results

showed that Cd-treated rats showed increase in activity of both GOT and GPT. Zn was found to significantly decrease activity of GOT and GPT. GSH estimation was conducted in tissue homogenates of liver to assess the burden of oxidative stress after treatment with Zn, Cd and their combination. The treatment of rats with Cd caused decrease in its level by 70.3%. Hitherto, group IV showed healing effect of Zn on Cd pre-treated rats demonstrating replenishment in GSH level by 80.7%. Cd-treated rats demonstrated increase in MDA level. In group IV, MDA level was found to be decreased by Zn indicating the ameliorative effect of Zn on Cd- toxicity. Treatment of rats with Zn caused a mild decrease in SOD activity as evidenced by group II but group III (Cd-treated rats) showed a marked decline in its activity by 45.2% in liver samples. Intriguingly, Zn showed a significant recovery in SOD activity by 45% as compared to group III. After the treatment with Cd, group III showed a decrease in Catalase activity as compared to the control. However, its activity was found to be recovered in group IV (Zn) in liver as compared to group III.

## Conclusion

Hence, Zn significantly induced oxidative stability in the hepatic tissues of a Cd-treated animal model.

